# Collagen type I PET/MRI enables evaluation of treatment response in pancreatic cancer in pre-clinical and first-in-human translational studies

**DOI:** 10.7150/thno.100116

**Published:** 2024-09-09

**Authors:** Shadi A. Esfahani, Hua Ma, Shriya Krishna, Sergey Shuvaev, Mark Sabbagh, Caitlin Deffler, Nicholas Rotile, Jonah Weigand-Whittier, Iris Y. Zhou, Ciprian Catana, Onofrio A. Catalano, David T. Ting, Pedram Heidari, Eric Abston, Michael Lanuti, Genevieve M. Boland, Priyanka Pathak, Hannah Roberts, Kenneth K. Tanabe, Motaz Qadan, Carlos Fernandez-del Castillo, Angela Shih, Aparna R. Parikh, Colin D. Weekes, Theodore S. Hong, Peter Caravan

**Affiliations:** 1 Athinoula A. Martinos Center for Biomedical Imaging, Department of Radiology, Massachusetts General Hospital, Harvard Medical School, Charlestown, MA, USA.; 2 Division of Nuclear Medicine and Molecular Imaging, Department of Radiology, Harvard Medical School, Massachusetts General Hospital, Boston, MA, USA.; 3 Institute for Innovation in Imaging, Department of Radiology, Massachusetts General Hospital, Harvard Medical School, Charlestown, MA, USA.; 4 Division of Hematology and Oncology, Department of Medicine, Massachusetts General Hospital Cancer Center, Harvard Medical School, Boston, Massachusetts, USA.; 5 Division of Thoracic Surgery, Massachusetts General Hospital Cancer Center, Harvard Medical School, Boston, Massachusetts, USA.; 6 Division of Gastrointestinal and Oncologic Surgery, Massachusetts General Hospital, Harvard Medical School, Boston, Massachusetts, USA.; 7 Department of Radiation Oncology, Massachusetts General Hospital, Harvard Medical School, Boston, Massachusetts, USA.; 8 Department of Pathology, Massachusetts General Hospital, Boston, Harvard Medical School, Massachusetts, USA.

**Keywords:** pancreatic cancer, type I collagen, PET imaging, treatment response, fibrosis

## Abstract

Pancreatic ductal adenocarcinoma (PDAC) is an invasive and rapidly progressive malignancy. A major challenge in patient management is the lack of a reliable imaging tool to monitor tumor response to treatment. Tumor-associated fibrosis characterized by high type I collagen is a hallmark of PDAC, and fibrosis further increases in response to neoadjuvant chemoradiotherapy (CRT). We hypothesized that molecular positron emission tomography (PET) using a type I collagen-specific imaging probe, ^68^Ga-CBP8 can detect and measure changes in tumor fibrosis in response to standard treatment in mouse models and patients with PDAC.

**Methods:** We evaluated the specificity of ^68^Ga-CBP8 PET to tumor collagen and its ability to differentiate responders from non-responders based on the dynamic changes of fibrosis in nude mouse models of human PDAC including FOLFIRNOX-sensitive (PANC-1 and PDAC6) and FOLFIRINOX-resistant (SU.86.86). Next, we demonstrated the specificity and sensitivity of ^68^Ga-CBP8 to the deposited collagen in resected human PDAC and pancreas tissues. Eight male participant (49-65 y) with newly diagnosed PDAC underwent dynamic ^68^Ga-CBP8 PET/MRI, and five underwent follow up ^68^Ga-CBP8 PET/MRI after completing standard CRT. PET parameters were correlated with tumor collagen content and markers of response on histology.

**Results:** ^68^Ga-CBP8 showed specific binding to PDAC compared to non-binding ^68^Ga-CNBP probe in two mouse models of PDAC using PET imaging and to resected human PDAC using autoradiography (P < 0.05 for all comparisons). ^68^Ga-CBP8 PET showed 2-fold higher tumor signal in mouse models following FOLFIRINOX treatment in PANC-1 and PDAC6 models (P < 0.01), but no significant increase after treatment in FOLFIRINOX resistant SU.86.86 model. ^68^Ga-CBP8 binding to resected human PDAC was significantly higher (P < 0.0001) in treated versus untreated tissue. PET/MRI of PDAC patients prior to CRT showed significantly higher ^68^Ga-CBP8 uptake in tumor compared to pancreas (SUV_mean_: 2.35 ± 0.36 vs. 1.99 ± 0.25, P = 0.036, n = 8). PET tumor values significantly increased following CRT compared to untreated tumors (SUV_mean_: 2.83 ± 0.30 vs. 2.25 ± 0.41, P = 0.01, n = 5). Collagen deposition significantly increased in response to CRT (59 ± 9% vs. 30 ± 9%, P=0.0005 in treated vs. untreated tumors). Tumor and pancreas collagen content showed a positive direct correlation with SUV_mean_ (R^2^ = 0.54, P = 0.0007).

**Conclusions:** This study demonstrates the specificity of ^68^Ga-CBP8 PET to tumor type I collagen and its ability to differentiate responders from non-responders based on the dynamic changes of fibrosis in PDAC. The results highlight the potential use of collagen PET as a non-invasive tool for monitoring response to treatment in patients with PDAC.

## Introduction

Pancreatic ductal adenocarcinoma (PDAC) is an aggressive cancer with a poor prognosis and a five-year overall survival of less than 9% 1, 2. Over the past two decades, the prevalence and mortality of PDAC have markedly increased and it is projected to become the second leading cause of cancer-related deaths before 2030 3. At the moment the only hope for cure is complete surgical resection, but only 10-15% of patients have resectable disease at presentation 4, 5. To increase the number of patients eligible for resection and improve outcomes, neoadjuvant chemoradiotherapy (CRT) has become the standard in patients with borderline resectable or locally advanced PDAC to downstage the tumor, expand surgical indications, and reduce nodal involvement 6-9. Chemotherapy regimens such as FOLFIRINOX (5-fluorouracil, leucovorin, irinotecan, and oxaliplatin), and gemcitabine plus nab-paclitaxel are commonly used to treat patients with metastatic disease. One of the major challenges in the management of PDAC is that standard imaging methods have poor reliability for the assessment of PDAC response to treatment because they do not accurately show the change in size or resectability of the residual tumor tissue 10-14. Although standard computer tomography (CT) or magnetic resonance imaging (MRI) is often performed at least two months apart, the changes in tumor size or morphology are not often precisely revealed 15. A large majority of patients need to undergo exploratory surgery and repetitive biopsies for post-treatment staging 10, 16, 17. Thus, there is an unmet need for the development of non-invasive imaging biomarkers to accurately monitor treatment response in PDAC.

Tumor fibrosis is a feature of PDAC caused by deposition of increased extracellular matrix (ECM), mainly type I collagen 18. While the fibrotic stroma in PDAC has often been proposed as a barrier to effective chemotherapy 18, 19, previous studies have shown that a positive response to neoadjuvant chemotherapy or CRT actually increases tumor fibrosis and is associated with improved outcomes in PDAC 14, 20. Therefore, measuring the change in tumor fibrosis could be used as a unique tool for PDAC response assessment to guide clinical management decisions and prognostication. Unfortunately, quantifying the temporal changes of tumor fibrosis is not possible with currently available conventional imaging modalities 10. A translatable molecular imaging tool is more suited to address this unmet need.

Our laboratory has developed 21, 22 and optimized a type I collagen specific positron emission tomography (PET) imaging probe, ^68^Ga-CBP8, and has validated it in multiple animal models of fibrotic diseases 21-24. This PET probe has recently been translated in healthy volunteers and patients with idiopathic pulmonary fibrosis and radiation induced lung injury 25-27. This study aims to develop a ^68^Ga-CBP8 PET image-guided paradigm for monitoring PDAC treatment response.

## Methods

### Study design

The overall goal of this study was to validate, optimize, and translate quantitative molecular imaging of tumor-associated fibrosis for non-invasive evaluation of treatment response in PDAC using a type I collagen PET imaging probe ^68^Ga-CBP8. We addressed these goals by 1) developing and selecting mouse models of human PDAC that were sensitive or resistant to standard-of-care FOLFIRNOX chemotherapy regimen, 2) determining the specificity of the ^68^Ga-CBP8 PET probe in binding to tumor collagen compared with a linear peptide non-binding control PET probe ^68^Ga-CNBP in untreated and in FOLFIRINOX- and vehicle-treated mouse models of PDAC over multiple treatment time points, 3) determining the optimal time of ^68^Ga-CBP8 PET acquisition in mouse tumor models, 4) confirming the ability and specificity of ^68^Ga-CBP8 to target collagen in human PDAC specimens *ex vivo* and lastly, 5) demonstrating the feasibility of ^68^Ga-CBP8 PET imaging for evaluation of response to standard neoadjuvant chemoradiotherapy in patients with PDAC. The animal experiments were approved and conducted per Institutional Animal Care and Use Committee (IACUC) guidelines, in accordance with ARRIVE guidelines (institutional protocol 2019N000139). Animals were randomly assigned to different groups. The human PDAC tumor and pancreas tissues were obtained from patients who underwent surgical resection at our institution and consented under a discarded tissue institutional protocol 02-240. The clinical feasibility study was performed in an open-label non-randomized fashion (patients consented and were recruited under protocol 2020P001899, NCT04485286). Pre-clinical and clinical PET image and histological analyses were performed blinded to the type of tumor, type of treatment, and imaging timepoint. The inclusion and exclusion criteria for the clinical study are detailed in **Table 1**. The sample size for each experiment and analysis method are mentioned in the results section and figure legends.

### *In vitro* cell culture, treatment, and viability assay

Human primary PDAC cells PANC-1 (ATCC), patient-derived metastatic cells PDAC6 (obtained from Dr. Ting's laboratory 28, and SU.86.86 (ATCC) were used. Cells were cultured in DMEM medium (for PANC-1 and PDAC6, ATCC) and RPMI (for SU.86.86, ATCC) supplemented with 10% fetal bovine serum (ThermoFisher) and 1% penicillin-streptomycin (ThermoFisher) at 37ºC and 5% CO_2_. Cells were tested for mycoplasma by PCR monthly.

A cell viability assay was performed to assess the level of sensitivity of the PDAC cells to FOLFIRINOX. Cells were cultured in 96-well plates with 100 µL of phenol-red-free medium. FOLFIRINOX drugs were dissolved in water to prepare stock solutions with 1000X concentration of each drug and then combined in a ratio similar to the routine clinical usage, with 1X defined as 3 μM 5-fluorouracil, 0.4 μM irinotecan, 0.3 μM oxaliplatin and 0.3 μM of calcium folinate (Sigma Aldrich) in the culture medium 28. When the cells reached approximately 90% confluency (5 x 10^3^ cells/well), FOLFIRINOX (0.001, 0.01, 0.1, 1, and 10 relative to 1X ratios) or vehicle (water) was added to the wells (3 wells per cell line and treatment group). Cell viability assay was performed at 24 and 48 h after treatment initiation using methods previously described 29. Briefly, the medium was removed, and cells were rinsed with phosphate buffered saline (PBS, Fisher Scientific). Subsequently, 10 µL of tetrazolium salt WST-1 (Sigma Aldrich) was added to 100 µL of the medium in each well. Cells were incubated for 30 min at 37 °C according to the manufacturer's protocol. The absorbance of the samples against a background control well as blank was measured at the wavelength of 450 nm using the BioTek Cytation-5 Imaging Reader (Winooski, VT, USA). The viability of each cell line was defined as the percentage of signal from the FOLFIRNOX-treated relative to the vehicle-treated wells.

### Mouse model of PDAC and FOLRINOX treatment

A total of 108 male athymic nude mice (Crl:NU(NCr)-Foxn1nu, strain 490, 6-8 week old, Charles River Laboratories, MA, USA) were used. Approximately 1-5 x10^6^ PDAC cells were mixed with Matrigel (BD Biosciences) in a total volume of 100 µL 1:1 (v/v) and injected in the subcutaneous space of the mice over the left shoulder 30. Tumor growth was monitored by calipers every 3 days and reported as volume = (π/6) x length x width^2^ (mm^3^) 31. When the tumors reached 100-250 mm^3^, mice were randomly assigned to the experimental groups.

Treatment was performed with tail vein injection of FOLFIRINOX (50 mg/kg leucovorin, 25 mg/kg fluorouracil, 25 mg/kg irinotecan, 5 mg/kg oxaliplatin dissolved in saline at a total volume of less than 150 µL) or vehicle (normal saline, less than 150 µL volume) every 3 days for up to a total of 8 treatment administrations 14.

### ^68^Ga-CBP8 and ^68^Ga-CNBP PET probe synthesis and biodistribution analysis

The collagen-binding peptide in CPB8 is a 16 amino-acid peptide with a 10 amino acid disulfide bridged cyclic component 21, 22. A linear control collagen non-binding peptide, CNBP was synthesized by reductive cleavage of the disulfide bond with the subsequent alkylation using iodoacetate as previously described 22. For the synthesis of ^68^Ga-CBP8 and ^68^Ga-CNBP PET probes, ^68^GaCl3 was eluted from a ^68^Ge/^68^Ga generator (Galli Eo TM, IRE-Elit, Fleurus, Belgium) with 1.1 mL of 0.1 mol/L hydrochloric acid, and ^68^GaCl_3_ (414 ± 55 MBq) was mixed with 100 µL of aqueous solution of CBP8 (or CNBP, 1 mmol/L) and 100 µL of sodium acetate buffer (pH 5.5, 1 M), and the mixture was heated to 95 °C for 15 min followed by cooling down at room temperature for 5 min. Sterile PBS and aqueous sodium hydroxide (1 M) were added to adjust the pH to 6.5-7 22. The radiochemical purity of the final solution was ≥ 99% for ^68^Ga-CBP8 and ≥ 97% for ^68^Ga-CNBP, as determined by radio-HPLC analysis (Agilent 1100 Series).

To study the biodistribution patterns of the collagen specific ^68^Ga-CBP8 and control ^68^Ga-CNBP probes, non-tumor-bearing male nude mice were injected with 4 MBq of either probe intravenously. At 1 h and 4 h post-injection, mice (16 total, n = 4 per probe type and time point) were euthanized and blood and different organs were extracted. The tissues were weighed and residual radioactivity in each was measured in the gamma counter (Wizard2, Perkin Elmer) and reported as the percentage of injected dose per gram (%ID/g).

### *In vivo* PET/MRI of PDAC animal models and PET data analysis

PET/MRI acquisition was performed using a Bruker Si 198 PET insert inside a Bruker 4.7T MRI scanner. Animals were anesthetized by inhalation of air/oxygen mixture and isoflurane (5% for induction and 1.5% for maintenance) via a face mask, and placed in a custom-built cradle with body temperature maintained at 37 °C. PET images were acquired in list mode from 0 to 60 min after tail vein injection of the ^68^Ga-CBP8 (11 - 20 MBq) or ^68^Ga-CNBP (11 - 15 MBq) in a single bed position. The MR images were acquired simultaneously for accurate anatomic localization. The sequences include T1-weighted Fast Low Angle Shot (T1w-FLASH) [Repetition time / Echo time / Flip angle = 21 ms/ 3 ms/ 12°, 0.4 mm isotropic spatial resolution, field of view = 85 mm × 65 mm] and T2-weighted Rapid Acquisition with Relaxation Enhancement (T2w-RARE) [Repetition time / Echo time / Flip angle = 1.4 s / 48 ms / 180°; resolution = 0.25 x 0.25 x 1 mm^3^, FOV 85 × 65 mm^2^]. Data was reconstructed using methods previously described 31. Images were analyzed using AMIDE software version 1.0.4. Three-dimensional regions of interest (ROI) were manually drawn over the tumors and left ventricle (as blood pool) guided by MRI. Standard PET uptake values were measured as the percentage of injected dosage per volume (%ID/cc).

### Evaluation of the specificity of ^68^Ga-CBP8 PET to tumor collagen and determination of the optimal imaging time

To assess the specificity of ^68^Ga-CBP8 to type I collagen in the tumor ECM, PANC-1 and PDAC6 tumor-bearing mice (n = 7-8/group) underwent dynamic PET imaging at 0 to 60 min after intravenous injection of ^68^Ga-CBP8 or ^68^Ga-CNBP probes (3.7-11 MBq, i.v.), in a random order over 2 consecutive days. The tumor uptake (%ID/cc) was compared between the two PET probes over time. At 30 min post-injection, the tumor-to-blood PET ratio reached above 1, and therefore, 30-60 min post-injection was determined as an optimal time for shorter static imaging in the subsequent experiments. After PET, the animals were euthanized and the tissues were harvested. The extracted tumor tissue slides were stained with Picro-Sirius Red (PSR), and tumor collagen content was correlated with the PET probe uptake for each probe type.

To confirm the specificity of ^68^Ga-CBP8 PET to changes in tumor collagen as compared to other factors such as a change in tumor vascular permeability over the course of treatment with chemotherapy, an additional group of PANC-1 tumor-bearing mice (n = 6) underwent PET imaging at 30-60 min after injection of ^68^Ga-CBP8 or ^68^Ga-CNBP probes prior to treatment, 9-10, and 15-16-days after treatment with FOLFIRINOX. Probes were injected in a random order on 2 consecutive days. The tumor PET probe uptake values were compared between the two probes on each day of the scan.

### Evaluation of the ability of ^68^Ga-CBP8 PET to monitor PDAC response to chemotherapy

Mice implanted with PDAC6, PANC-1, or SU.86.86 cells were randomized into 2 groups for treatment with FOLFIRINOX or vehicle every 3 days for up to 24 days. Animals in each group underwent PET/MRI with ^68^Ga-CBP8 at baseline, 9- and 15-days post-treatment (n = 4-8/group). Tumor growth was monitored every 3 days for a total of 24 days. Tumor PET uptake values and growth curves were compared among multiple time points for each tumor type and treatment group.

### *Ex vivo* evaluation of ^68^Ga-CBP8 Probe for quantification of human tumor fibrosis by autoradiography

Fresh resected excess human tumors and adjacent pancreas tissues were obtained. The tissues were cut into small pieces of 30-50 μm thickness and rinsed with sterile PBS at room temperature. Each tissue was incubated with 0.5 MBq of either ^68^Ga-CBP8 or the control ^68^Ga-CNBP probe on a rotating shaker for 30 min. Following three times of rinses with PBS for a total of 3 min, the tissues were allowed to dry at room temperature. The radioactivity within the tissues was evaluated on an autoradiography system (Perkin Elmer). On the following day, the same protocol was performed by incubation of the same tissues with the other probe type. Acquired images were analyzed using ImageJ software (version 1.5.3, NIH, Bethesda, USA) by drawing an ROI over the tissues and recording a mean arbitrary signal.

### Feasibility evaluation of ^68^Ga-CBP8 PET/MRI for assessment of treatment response in patients with PDAC

Study participants with biopsy-proven borderline resectable or locally advanced PDAC were consented from July 2021 to March 2023 and enrolled in the study if the criteria were met **(Table 1)**. Patients underwent a baseline ^68^Ga-CBP8 PET/MRI of the upper abdomen prior to starting neoadjuvant treatment and after completion of the standard-of-care CRT including FOLFIRINOX followed by short (10 fractions) or long-term (28 fractions) radiation and capecitabine 32. Dynamic PET imaging of the upper abdomen was performed for up to 90 min after intravenous (i.v.) injection of the ^68^Ga-CBP8 using a Siemens mMR Biograph 3 Tesla MR/PET scanner. Simultaneous MR images including T1-, T2-, and diffusion-weighted images and multi-phase (including late arterial, portal venous, and delayed phase) contrast-enhanced images of the pancreas were obtained following administration of gadoterate meglumine (0.2 mL/kg) at an injection rate of 2 mL/s, using the institutional standard protocol with a total acquisition time of up to 25 min. PET images were reconstructed after applying the parameters for detector efficiency, decay, dead time, scatter, and MR-based attenuation correction. Image processing and analysis were performed by an abdominal radiologist and a nuclear radiologist with 15 and 8 years of experience in the analysis and interpretation of PET and MRI. The study investigators were blinded to disease status (pre- vs. post-CRT) using the same tools (AMIDE software) to minimize any technique-related variability. Guided by the MR images, an ROI was drawn over the pancreatic tumor, un-involved pancreas, and the descending aorta at the level of the tumor (as blood pool). PET standard uptake values (SUV_mean_) of the tumor, pancreas, and blood were calculated over time and compared between the pre-and post-CRT PET scans. The SUV_mean_ in the tumors and pancreas on pre- and post-CRT PET scans were correlated with the collagen content of diagnostic core and resected tissues.

Patients' demographics including age, gender, race, ethnicity, Eastern Cooperative Oncology Group (ECOG) performance status, initial local staging of the tumor, location of the primary tumor in the pancreas, CA-19-9 level prior to treatment and prior to surgery, neoadjuvant treatment regimen, and type of surgery were reported.

### Histological analyses

The extracted tumor and pancreas tissues were fixed in formalin and the paraffin-embedded tissue blocks were cut into 5 μm thick slides. The tissues were stained with hematoxylin and eosin (H&E), Masson Trichrome, and PSR. Immunohistochemical (IHC) staining was performed using methods previously described 31. The slides were incubated with mouse monoclonal primary antibody for collagen type I (ab88147, Abcam) at 1/50 dilution followed by goat anti-mouse HRP conjugated secondary antibody (ab6789, Abcam) at 1/1000 dilution, and with rabbit monoclonal primary antibody for cleaved caspase-3 (9579S, Cell Signaling) at 1/500 dilution, followed by goat anti-rabbit HRP-conjugated secondary antibody (ab6721) at 1/1000 dilution. The slides were then mounted with Prolong Gold Antifade Reagent and DAPI (ab8961, Abcam). Microscopic evaluation was performed by BioTeK Citation 5 microscopy system. Biomarker quantification was performed using ImageJ software (version 1.5.3.0). Collagen was quantified as collagen proportion area (CPA) defined as the percentage of positive collagen staining over the total tissue area. Cleaved caspase-3 was reported as the percentage of positive staining relative to the total cell number.

For the patients under the feasibility study, slides for the diagnostic core samples (1 H&E and 3 slides stained with PSR) and resected tumor tissues with adjacent un-involved pancreas, if available (1 H&E and 1 PR-stained slide per block, 3-31 blocks per tissue) were obtained. The mean tumor and adjacent pancreas CPA were calculated and compared between the pre- and post-treatment samples. The tissue CPA was correlated with the tissue SUV_mean_ on the PET.

### Hydroxyproline assay

Part of the mouse PDAC and pancreas tissues were immediately flash frozen in liquid nitrogen for measuring Hyp and Pro by high-performance liquid chromatography (HPLC) analysis using an established method and was recorded as amounts per wet weight of the tissue 22. Collagen content was quantified and reported as the ratio of Hyp/Pro.

### Statistical analysis

Statistical analysis was performed using GraphPad Software (version 10, CA, USA). Continuous variables are presented as mean ± standard deviation (SD). Two-tailed paired Student's t-test was performed to compare the means of one group under two different conditions and an unpaired t-test was performed to compare the means of two different groups. ANOVA with multiple comparisons and post hoc Tukey's test was used to compare the mean values among multiple groups. ANOVA with repeated measures was performed to compare the mean values for the same mice over multiple time points. Pearson's correlation coefficient was calculated to examine the relationship between the PET probe uptake values and tissue collagen content (CPA). A P-value of less than 0.05 was considered statistically significant.

## Results

### *In vitro* viability assay

**Figure 1:**
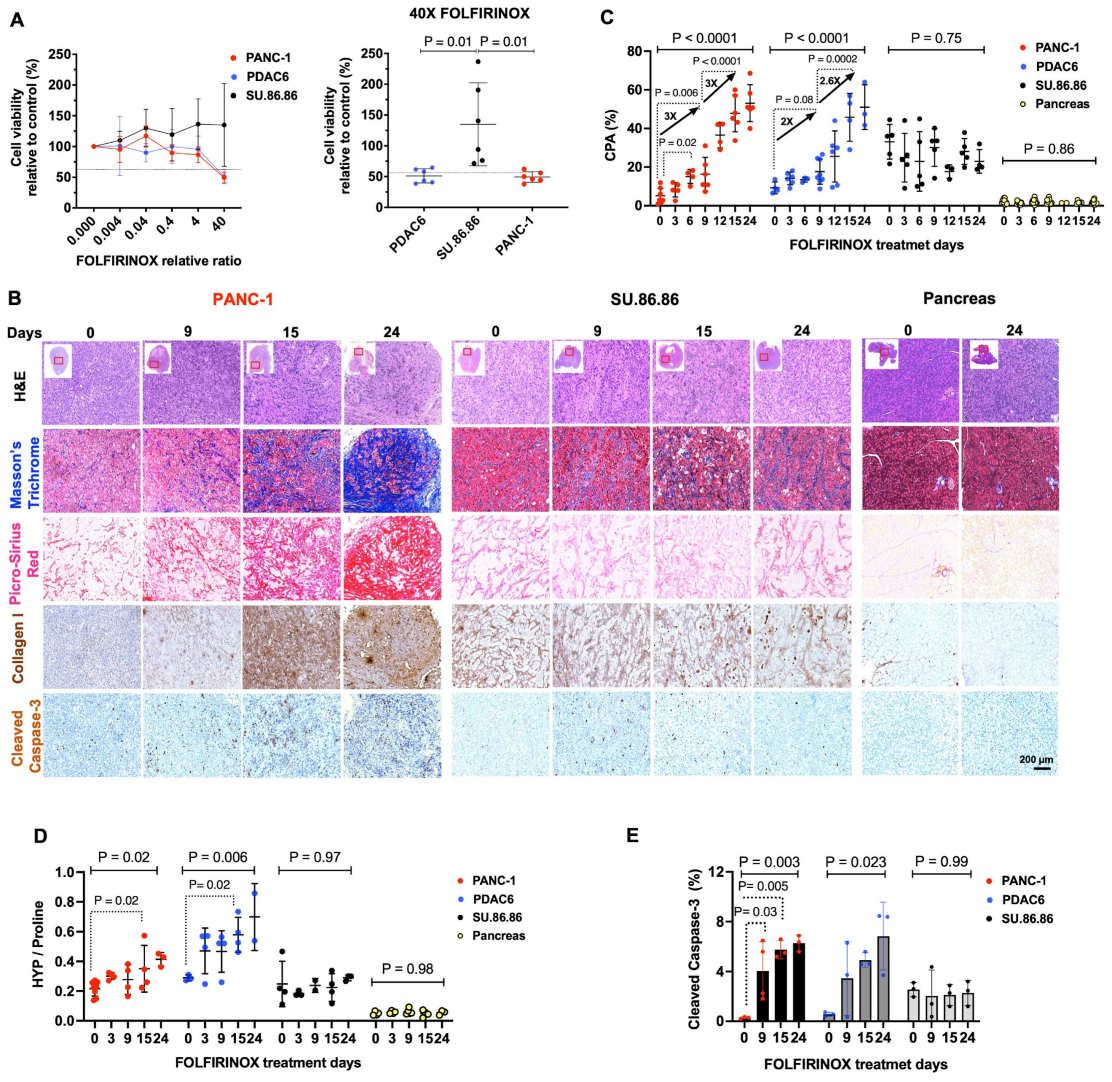
** Collagen increases in the tumor extracellular matrix in response to chemotherapy in mouse models of human PDAC. (A)** Cell viability assay at various concentrations of FOLFIRINOX chemotherapy demonstrating the sensitivity of PDAC6 and PANC-1 cells at 24- and 48-h post-treatment and the relative resistance of SU.86.86 cells under the same conditions (n = 3-6 wells/condition). **(B)** Representative histological evaluation of pancreas and subcutaneously grown tumor tissues in the nude mice extracted before and at multiple days after treatment with FOLFIRINOX (administered every 3 days) and stained with hematoxylin and eosin (H&E), Masson Trichrome and Picro-Sirius Red (PSR) for collagen, immunohistochemical staining for type I collagen, and cleaved caspase-3 for apoptosis (Scale bar: 200 µm). **(C)** Quantitative analysis of collagen from PSR stained tissue reported as collagen proportional area (CPA). **(D)** Measure of collagen on the extracted tumors reported as hydroxyproline-to-proline (Hyp/Pro) ratio. **(E)** Quantitative immunohistochemical staining of apoptosis marker cleaved caspase-3 in the tumors at multiple time points. Each data point represents one mouse. Data are shown in mean ± SD. P values are the result of one-way ANOVA with multiple comparisons and post hoc Tukey test.

Treatment of different PDAC cell lines with different concentrations of FOLFIRINOX demonstrated that PANC-1 and PDAC6 cells had a higher sensitivity to FOLFIRINOX, assessed by the concentration required to kill half the cells with a 48-h incubation, while SU.86.86 cells were resistant to FOLFIRINOX under the same conditions **(Figure 1A)**.

### Collagen deposition continuously increases in PDAC in response to FOLFIRINOX

Mouse models of subcutaneously engrafted PDAC were treated with i.v. FOLFIRINOX every 3 days. Tumors and pancreas were extracted prior to, and at 3, 6, 9, 12, 15, or 24 days after starting the treatment (n = 3-8/tumor type and time point). Microscopic evaluation of the extracted PANC-1 and PDAC6 tumors demonstrated an overall decrease in cellularity in H&E stained tissue and increased cleaved caspase-3 marker of apoptosis over 24 days of FOLFIRINOX treatment confirming response. A continuous accumulation of collagen within the tumor ECM was observed in Masson's trichrome, PSR, and Collagen type I IHC stained tissue. The cellularity, apoptosis marker, or collagen deposition did not change in resistant SU.86.86 tumors or normal extracted pancreas tissues **(Figure 1B, Figure S1A)**. The visual assessment of fibrotic response in tissues was confirmed by quantitative evaluation of collagen by measuring CPA in PSR-stained slides and the Hyp/Pro ratio. In PANC-1 tumors, the CPA started to show a significant increase on day 6 compared to baseline (CPA: 5 ± 4% vs. 15 ± 4%, at day 0 vs. day 6, respectively, P = 0.02), with an approximately threefold increase from baseline to day 9 (CPA: 5 ± 4% vs.16 ± 9%, P = 0.006) and a threefold increase from day 9 to day 24 (CPA: 16 ± 9% vs. 53 ± 10%, P < 0.0001) **(Figure 1C)**. In PDAC6, the same overall trend was shown although with a slightly slower fibrotic response compared to PANC-1; the CPA increased twofold on day 9 compared to baseline (CPA: 9 ± 3% vs.18 ± 6%, at day 0 vs. day 9, respectively, P = 0.08) and 2.6 fold from day 9 to day 24 (CAP: 18 ± 6% vs. 51 ± 11 P = 0.0002) **(Figure 1C)**. There was no change to slightly decreased CPA in the FOLFIRINOX resistant SU.86.86 tumors (CPA: 33 ± 9% vs. 23 ± 6% at day 0 vs. day 24 respectively, P = 0.75) and unchanged minimal collagen within the pancreas (CPA: 2 ± 1% vs. 2 ± 1% at day 0 vs. day 24, respectively, P = 0.86) **(Figure 1C)**. The CPA results were confirmed by HPLC analyses for hydroxyproline (Hyp) and proline (Pro) in the tissues. There was an overall increase in the Hyp/Pro ratio in PANC-1 and PDAC6 tumors over 24 days of treatment (n = 3-8 per tumor models and timepoint, Hyp/Pro at day 0 vs. day 24 for PANC-1: 0.22 ± 0.05 vs. 0.42 ± 0.05, P = 0.02; for PDAC6: 0.29 ± 0.02 vs. 0.70 ± 0.22, P = 0.006) and no significant change in Hyp/Pro in SU.86.86 tumors or normal pancreas over 24 days of treatment (P = 0.97 and 0.98, respectively, one-way ANOVA with multiple comparisons and post hoc Tukey test) **(Figure 1D)**. The level of response to treatment was confirmed by IHC with a significant increase in cleaved caspase-3 in the PANC-1 and PDAC6 and no significant change in SU.86.86 tumors (P = 0.003, 0.023, and 0.99, respectively, one-way ANOVA with multiple comparisons and post hoc Tukey test) over 24 days **(Figure 1E)**. Based on these histological and biochemical results, day 9 (after 3 doses of FOLFIRINOX) and day 15 (after 5 doses of FOLFIRINOX) were selected as the time points for *in vivo* PET imaging experiments in this study.

### ^68^Ga-CBP8 PET probe specifically binds to tumor collagen in animal models of PDAC

**Figure 2:**
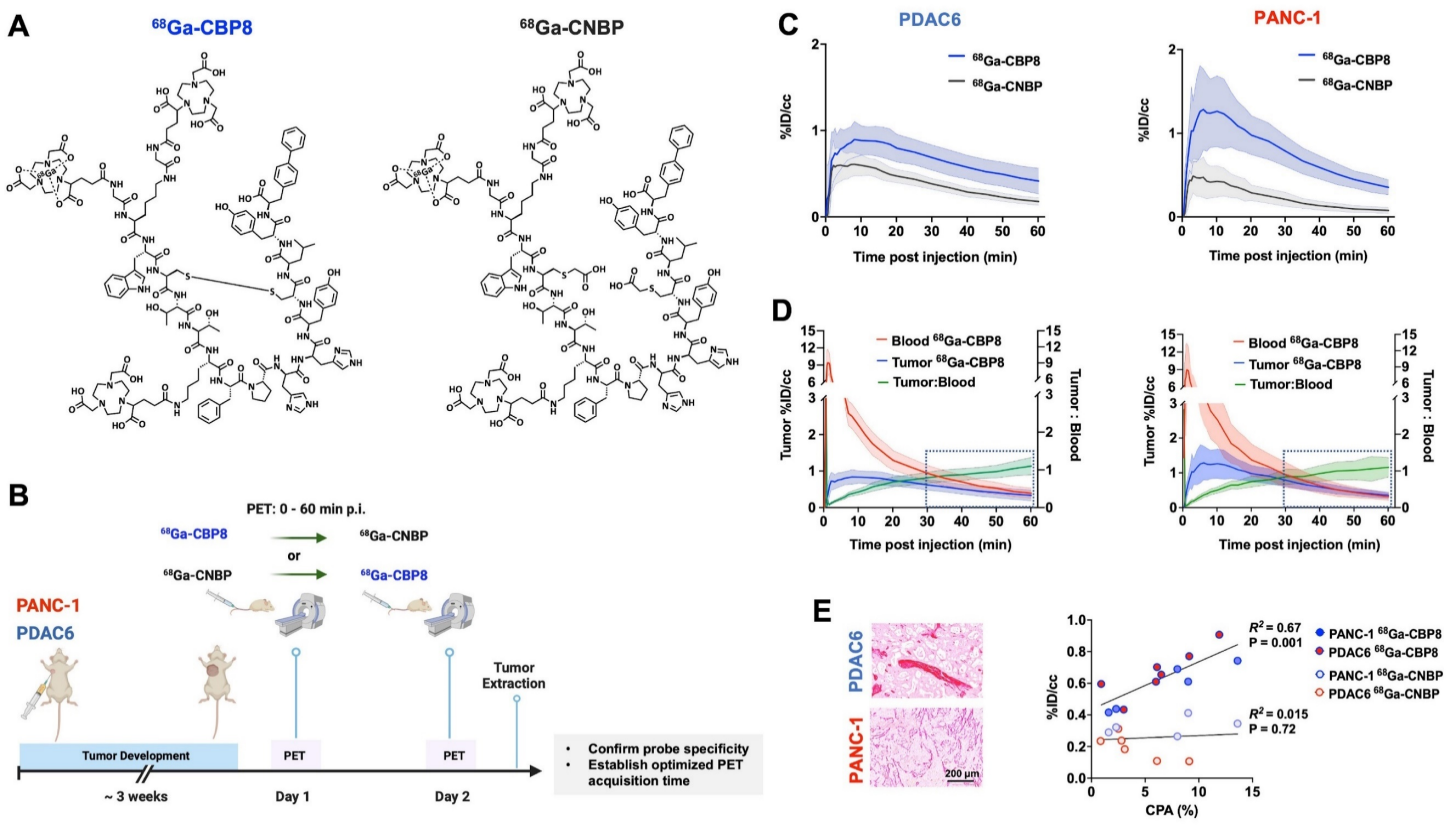
**^68^Ga-CBP8 specifically binds to collagen type 1 in mouse models of PDAC. (A)** Structure of the type I collagen binding ^68^Ga-CBP8 and control nonbinding ^68^Ga-CNBP probes. There are 3 NODAGA moieties for potential Ga-68 labeling and the radiolabeled product is a mixture of these three isomers with a representative isomer shown here. **(B)** Schematic illustration of the study design for testing the specificity of ^68^Ga-CBP8 and control ^68^Ga-CNBP probes in untreated PDAC models based on dynamic PET over two consecutive days. **(C)** Tumor uptake curves from dynamic PET acquired 0-60 min after intravenous injection of each probe (^68^Ga-CBP8 in blue line and ^68^Ga-CNBP in black line) quantified as the percentage of injected dosage per cc (%ID/cc) (n = 4-7 male nude mice, Data are shown in mean ± SD). **(D)** Clearance of the ^68^Ga-CBP8 from the blood (red line) over 60 min and tumor-to-blood uptake ratio (green line) reaching above 1 at 30-60 min post-injection (n = 5-6, Data are shown in mean ± SD). **(E)** Representative Picro-Sirius Red stained tissue of extracted tumors and correlation of collagen proportional area (CPA) with the mean tumor PET uptake values at 30-60 min of imaging with the specific ^68^Ga-CBP8 (R^2^ = 0.67, P = 0.001) and linear control ^68^Ga-CNBP (R^2^ = 0.015, P = 0.72) (each data point represents one mouse). PANC-1 data is shown in orange and PDAC6 in blue color.

The structure of collagen binding probe ^68^Ga-CBP8 and collagen non-binding, linear peptide control probe ^68^Ga-CNBP are shown in **Figure 2A.** *Ex vivo* biodistribution analyses in a group of non-tumor-bearing mice demonstrated rapid renal clearance of both probes by 1 h post-injection **(Figure S2A)**. Residual radioactivity from ^68^Ga-CBP8 was significantly lower than ^68^Ga-CNBP in lung, kidney, and liver (P < 0.05) and not significantly different in blood and other organs. At 4 h post-injection, there was further elimination of both probes from the blood and normal organs with no significant difference in their overall biodistribution pattern (P > 0.05 for different organs) **(Figure S2B)**.

**Figure 3:**
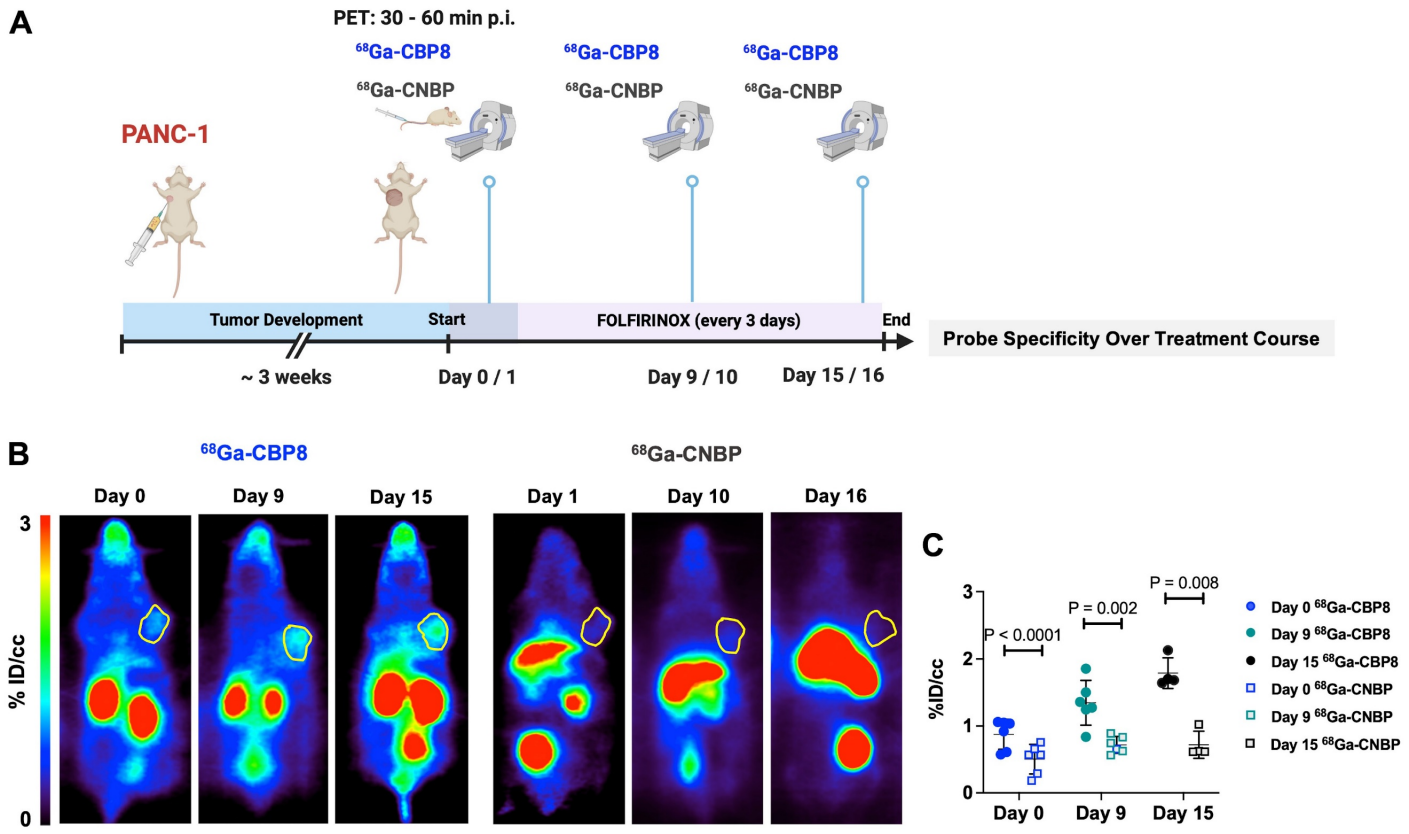
**^68^Ga-CBP8 shows specific changes in type I collagen in response to chemotherapy in mouse models of PDAC. (A)** Schematic illustration of study design to demonstrate the specificity of ^68^Ga-CBP8 compared to control ^68^Ga-CNBP PET over multiple days of treatment with FOLFIRINOX (administered every 3 days) in PANC-1 tumor-bearing mice (male nude mice, n = 4-6/group). **(B-C)** Representative coronal PET images (tumors shown in yellow circle) and quantitative analyses demonstrating a significantly higher uptake of ^68^Ga-CBP8 in the tumors at all time points compared to the control ^68^Ga-CNBP (paired t-test, two-tailed), highlighting the specificity of the probe for collagen I. Data also show an increase in tumor ^68^Ga-CBP8 following FOLFIRINOX treatment but no increase in ^68^Ga-CNBP tumor uptake with treatment. Data are shown in mean ± SD.

A schematic illustration of the study design is shown in **Figure 2B**. Dynamic PET imaging of PANC-1 or PDAC6 tumor-bearing mice was performed on consecutive days. On day 1 ^68^Ga-CBP8 or the control probe ^68^Ga-CNBP was administered intravenously, and the next day the other probe was used; the order of probe administration was randomized. There was significantly higher ^68^Ga-CBP8 tumor uptake compared to ^68^Ga-CNBP at 0-60 min post-injection (P < 0.05, paired t-test). The tumor-to-blood uptake ratio of ^68^Ga-CBP8 exceeded 1 by 30 min post-injection and the 30-60 min data collection time interval was chosen for subsequent PET imaging experiments **(Figure 2C-D, Figure S3)**. There was a significant positive correlation between tumor collagen content (CPA) and tumor ^68^Ga-CBP8 PET uptake values, whereas no significant correlation was found with the ^68^Ga-CNBP (R^2^ = 0.67, P = 0.001, and R^2^ = 0.015, P = 0.72, respectively) **(Figure 2E)**. In a separate group of mice engrafted with PANC-1 tumors, PET imaging with ^68^Ga-CBP8 and ^68^Ga-CNBP was performed on two consecutive days at baseline, 9/10, and 15/16 days after treatment with FOLFIRINOX **(Figure 3A)**. There was a significantly higher tumor uptake with ^68^Ga-CBP8 compared with ^68^Ga-CNBP at each time point (P < 0.05, paired t-test). While the tumor uptake with ^68^Ga-CBP8 increased over time (tumor %ID/cc on day 0, 9, 15: 0.87 ± 0.22, 1.35 ± 0.33, 1.79 ± 0.23, P = 0.0006, one-way ANOVA with post hoc test), the ^68^Ga-CNBP PET uptake values did not significantly change (tumor %ID/cc on day 0, 9, 15: 0.50 ± 0.22, 0.71 ± 0.12, 0.71 ± 0.20, P = 0.77, one-way ANOVA) **(Figure 3B-C)**.

### ^68^Ga-CBP8 PET enables non-invasive monitoring of response to chemotherapy in PDAC mouse models

**Figure 4:**
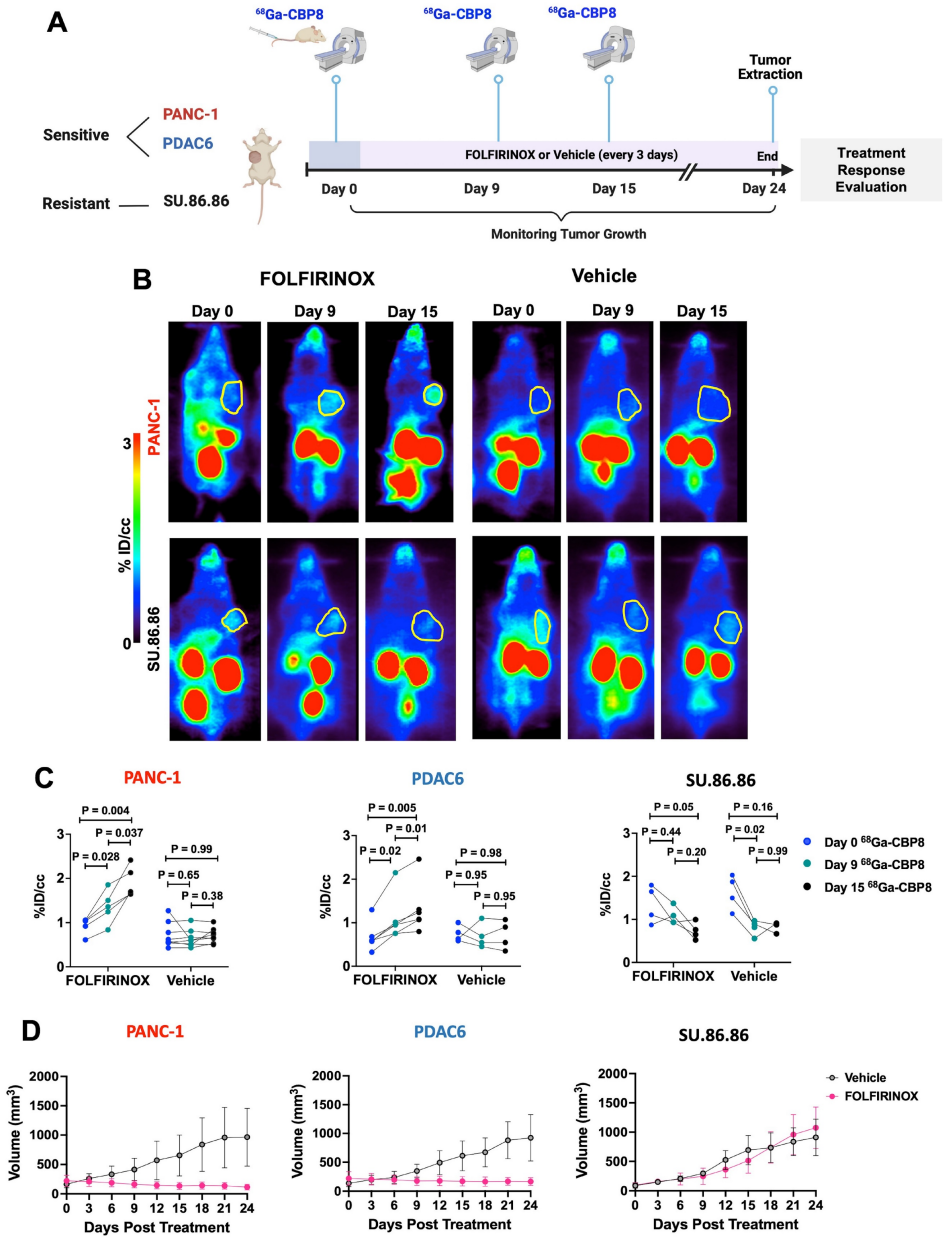
**^68^Ga-CBP8 enables monitoring of the response to chemotherapy in mouse models of human PDAC and differentiates responders from non-responders. (A)** Schematic study design. **(B)** Representative coronal PET images of the nude mice with PANC-1 and SU.86.86 tumors imaged from 30-60 min after injection of ^68^Ga-CBP8 at days 0, 9, and 15 after treatment with intravenous FOLFIRINOX or vehicle (tumors shown in yellow circle). **(C)** Quantitative analyses of ^68^Ga-CBP PET tumor uptake at days 0, 9, and 15 of treatment with FOLFIRINOX or vehicle (n = 4-8/group, ANOVA with multiple comparisons and post hoc Tukey test). **(D)** Tumor volume curves over 24 days of treatment with FOLFIRINOX or vehicle. Data are shown in mean ± SD.

^68^Ga-CBP8 uptake values continuously increased in response to duration of FOLFIRNINOX treatment in FOLFIRINOX-sensitive PANC-1 tumors (%ID/cc: 0.8 ± 0.2 vs. 1.1 ± 0.3 vs. 1.9 ± 0.2, in day 0 vs. day 9 vs. day 15, respectively, P < 0.05 among time points, ANOVA with multiple comparisons, n = 5), and FOLFIRINOX-sensitive PDAC6 tumors (%ID/cc: 0.68 ± 0.32 vs. 1.1 ± 0.52 vs. 1.32 ± 0.58, n = 6, P < 0.05 among time points, ANOVA with multiple comparisons). On the other hand, no significant change was observed in ^68^Ga-CBP8 PET tumor values in vehicle treated or in FOLFIRINOX treated resistant SU.86.86 tumors among different time points (P > 0.05, n = 4-8/group) **(Figure 4A-C, Figure S4)**. There was a significant tumor growth reduction in PANC-1 and PDAC6 tumors in response to FOLFIRINOX compared to their vehicle treated groups, but not for SU.86.86 tumors (day 24 tumor volume mm^3^ for FOLFIRINOX vs. vehicle: 116 ± 49 vs. 964 ± 491, P = 0.0116 for PANC-1, 183 ± 66 vs. 936 ± 403, P = 0.0011 for PDAC6, and 909 ± 311 vs. 1074 ± 354, P = 0.39 in SU.86.86, unpaired t-test) **(Figure 4D)**.

### ^68^Ga-CBP8 specifically binds to human PDAC *ex vivo*

**Figure 5:**
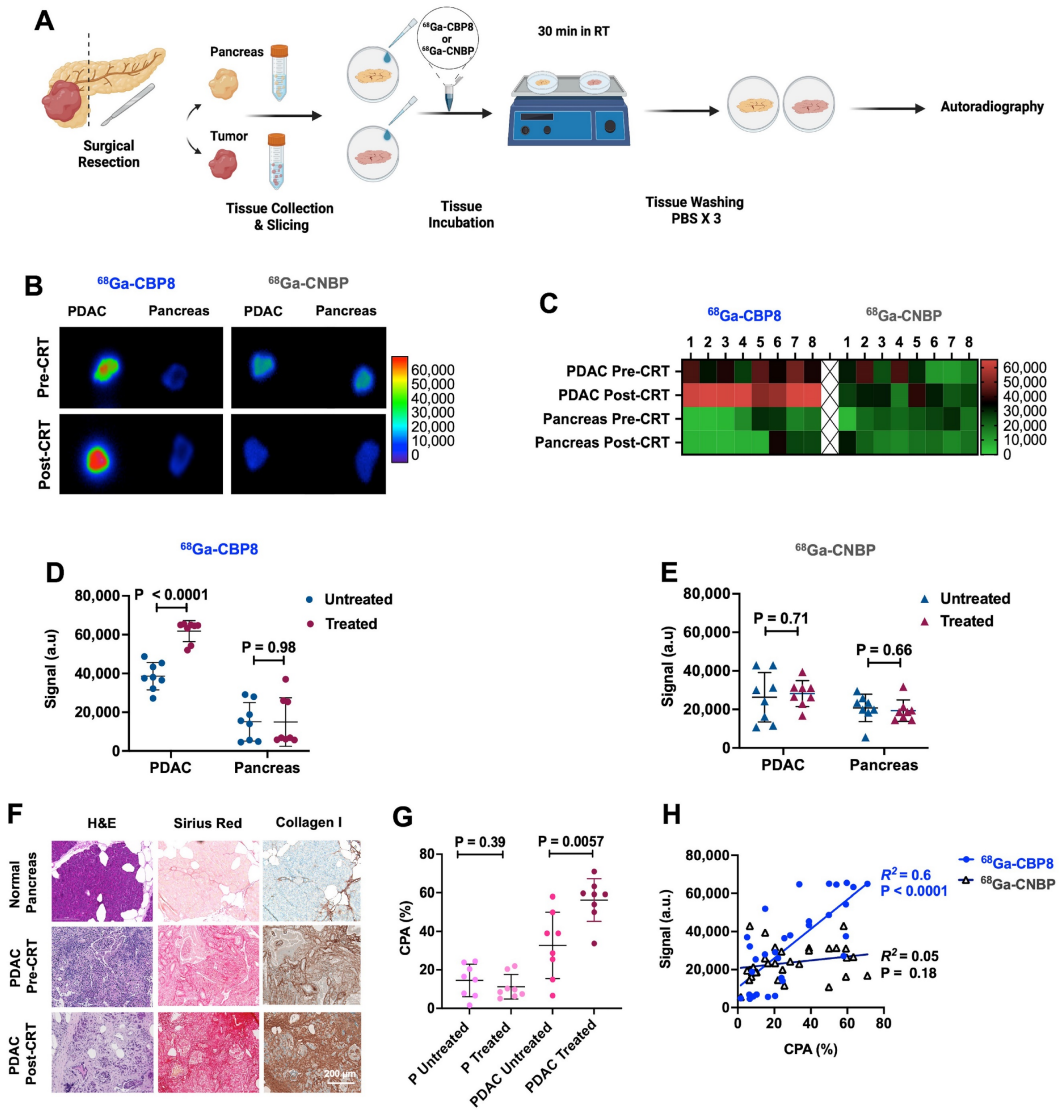
**^68^Ga-CBP8 detects fibrosis and measures response to neoadjuvant chemoradiotherapy in resected human pancreatic tumor tissues. (A)** Schematic illustration of human pancreatic ductal adenocarcinoma (PDAC) and pancreas tissue preparation, incubation with collagen-specific ^68^Ga-CBP8 and control ^68^Ga-CNBP probes, and autoradiography. **(B)** Representative autoradiography images of fresh resected human tissues and **(C)** heatmap of the autoradiography signal. **(D-E)** Quantitative comparison of autoradiography signal in the treated and untreated PDAC tissues and the patients' matched adjacent uninvolved pancreas (P) for ^68^Ga-CBP8 **(D)** and ^68^Ga-CNBP **(E)** (n = 8/group, unpaired t-test, two-tailed). **(F)** Representative H&E, Picro-Sirius Red (PSR), and type I collagen immunohistochemical staining of human tissues (scale bar: 200 µm). **(G)** Quantitative comparison of collagen proportional area (CPA) from PSR stained slides of human tumor and pancreas tissues (n = 8/group, unpaired t-test, two-tailed). **(H)** Correlation of the ^68^Ga-CBP8 or ^68^Ga-CNBP autoradiography signal with CPA. Each data point represents one tissue. RT: room temperature, a.u: arbitrary unit.

Surgically resected tissues from patients who completed standard 8 cycles of pre-operative FOLFIRINOX followed by CRT (n = 8, M/F: 3/5, age: 56 - 82 y) and patients who underwent upfront resection of their PDAC (n = 8, M/F: 3/5, age: 58 - 78 y) were incubated with ^68^Ga-CBP8 or ^68^Ga-CNBP and assessed by autoradiography **(Figure 5A)**. There was significantly higher ^68^Ga-CBP8 binding in treated compared to untreated PDAC tissues (P < 0.0001, unpaired t-test) and when compared to each patient's matched normal pancreas (P < 0.0001, unpaired t-test). No significant difference in ^68^Ga-CBP8 was observed between the normal uninvolved pancreas tissues of treated and untreated patients (P = 0.98, unpaired t-test) **(Figure 5B-D)**. The control probe ^68^Ga-CNBP was not sensitive to tumor collagen in either group of tissues and the retained activity was not significantly different between the treated and untreated tissues (P = 0.71 and 0.66 in untreated vs. treated PDAC and pancreas tissues respectively) **(Figure 5B,C,E)**. Histological evaluation of the tissues showed an increased collagen deposition in the tumors in response to CRT (CPA: 56.3 ± 11% vs. 31.7 ± 17.2%, P = 0.0057 in treated vs. untreated PDAC respectively), while it remained low and not significantly changed in the adjacent pancreas tissues between the treated and untreated tissues (CPA: 11.2 ± 6.4% vs. 14.5 ± 8.4%, P = 0.39) **(Figure 5F-G)**. A significant positive correlation was noted between ^68^Ga-CBP8 signal in autoradiography and the amount of tumor or pancreas collagen assessed by CPA (R^2^= 0.6, P < 0.0001), while no such correlation was observed with ^68^Ga-CNBP (R^2^= 0.05, P = 0.18) **(Figure 5H)**. Together these results confirm the specificity of ^68^Ga-CBP8 to type I collagen in human PDAC.

### ^68^Ga-CBP8 PET/MRI of PDAC patients

Eight patients (all male, white non-Hispanic, mean age ± SD: 60 ± 5 y, range: 49 - 65 y) with newly diagnosed PDAC (6 with borderline resectable and 2 with locally advanced PDAC) were enrolled in this feasibility study. Patients underwent dynamic PET for 47-90 min after administration of ^68^Ga-CBP8 probe (122-281 MBq) followed by standard contrast-enhanced MRI of the pancreas prior to starting the neoadjuvant treatment. The first patient declined the repeat pre-operative scan, and the fifth patient was unable to return for repeat scan due to an accelerated surgery schedule. The last patient showed local progression of the primary tumor and received additional cycles of FOLFIRINOX and gemcitabine/nab-paclitaxel chemotherapy therefore surgery was not planned. Five (5/8) patients underwent repeat PET/MRI after completion of CRT (repeat PET duration: 30 - 90 min, administered dosage:128 - 220 MBq). All patients tolerated the scan and administration of the radiopharmaceutical without any adverse effects. The CA-19-9 level decreased in all the patients during the treatment, and tumors decreased in size as measured by standard CT scans before and after the completion of treatment. Two patients received losartan during the treatment; one of which showed vascular involvement of the primary tumor at laparotomy and the other one showed fibrosis without any evident live tumor on the intra-operative biopsy of the tumor. These two patients underwent intra-operative radiation followed by gastrojejunostomy. The remaining patients underwent the Whipple procedure **(Table S1 and Table S2)**.

**Figure 6:**
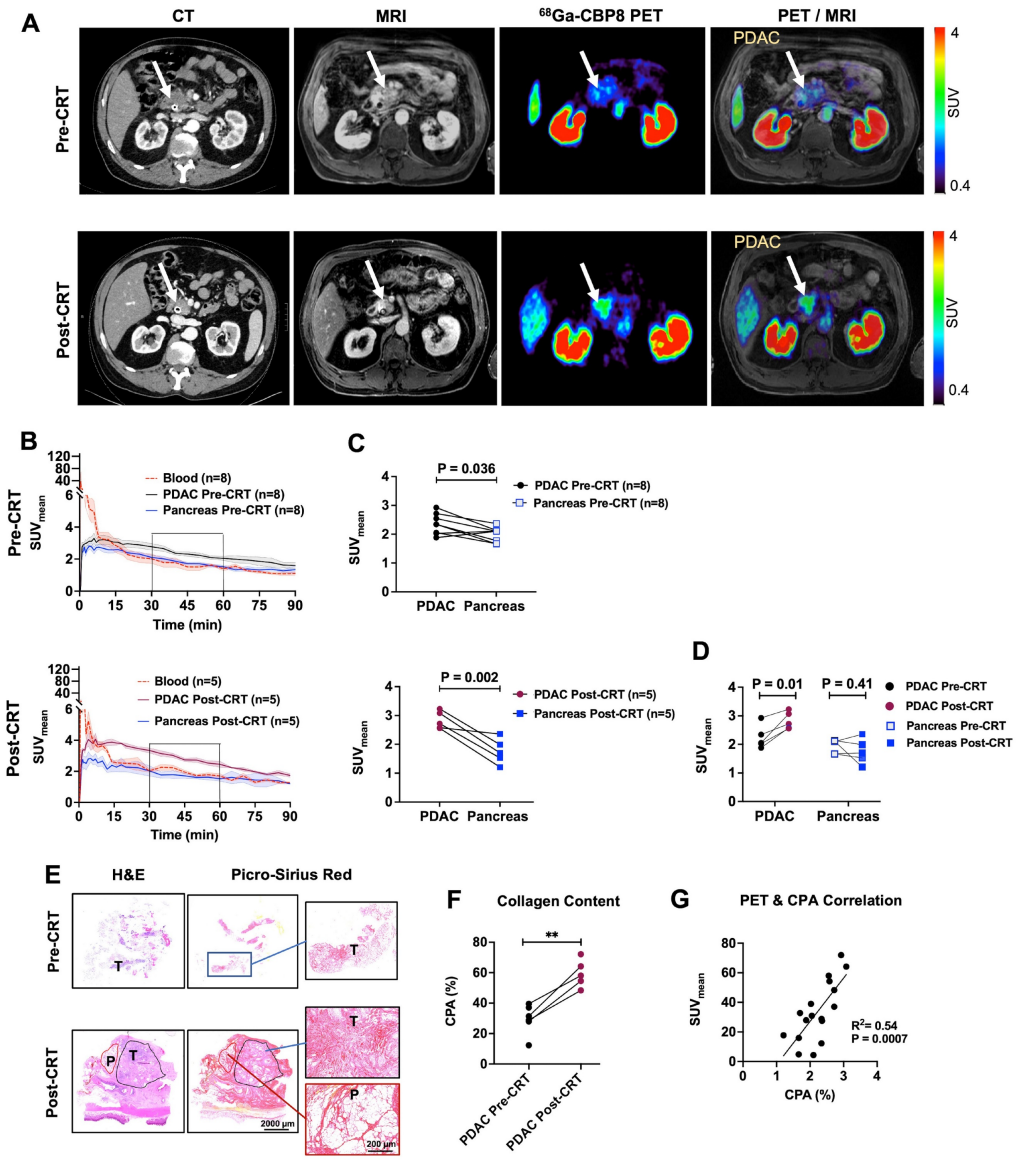
** First-in-human evaluation of type I collagen PET in patients with PDAC. (A)** Representative axial images of standard-of-care iodinated contrast-enhanced computed tomography (CT) on late arterial phase, gadolinium-enhanced magnetic resonance image (MRI) on portal venous phase, ^68^Ga-CBP8 PET at 30-45 min post-injection, and fused PET and T1-weighted MR images in the axial plane are shown for a subject before and after standard chemoradiotherapy (CRT). PDAC tumor is shown with a white arrow. **(B)** Evaluation of dynamic PET from 0-90 min after injection of the ^68^Ga-CBP8 shows rapid clearance of the probe from the blood (dashed red line), with blood SUV_mean_ reaching that of the uninvolved pancreas SUV_mean_ (blue line) at 15-30 min post-injection. Uptake to untreated PDAC is shown with black and to treated PDAC with dark red line. Data is shown in mean ± SEM. **(C)** The tumor SUV_mean_ is significantly higher than the pancreas at both pre-CRT and post-CRT scans (paired t-test). **(D)** PET uptake values at 30-60 min post-injection for the five patients who underwent PET scans at both time points show increased tumor SUV_mean_ in response to neoadjuvant CRT paired t-test). **(E)** Representative H&E and Picro-Sirius Red staining of the diagnostic core of tumor (T), and resected treated tumor and adjacent pancreas (P) tissues. **(F)** Quantitative analyses of histology show higher CPA in the treated compared to untreated tumors (** P = 0.0005 for all available tissues using unpaired t-test; P = 0.005 for 4 patients with available pre- and post-CRT tissues using paired t-test). **(G)** Significant positive correlation between the tissue collagen on histology (CPA) and SUV_mean_.

Representative standard CT and ^68^Ga-CBP8 PET/MRI of a patient pre- and post-CRT are shown in** Figure 6A**. PET image analyses at both pre-CRT and post-CRT visits showed rapid renal clearance of the ^68^Ga-CBP8 probe from the blood and normal pancreas. By 15-30 min, the blood SUV_mean_ reached the value of the uninvolved pancreas SUV_mean_ **(Figure 6B)**. The tumor SUV_mean_ remained significantly higher than the pancreas at both pre-CRT (mean SUV_mean_ at 30-60 min: 2.35 ± 0.36 vs. 1.99 ± 0.25, P = 0.036, unpaired t-test, two-tailed) and post-CRT scans (2.83 ± 0.30 vs. 1.75 ± 0.43, P = 0.002, unpaired t-test, two-tailed) **(Figure 6C)**. In the five patients who underwent a repeat PET/MRI scan, the tumor was reduced in size on concurrent MRI and the PET probe uptake values significantly increased in the treated compared to untreated tumors (SUV_mean_: 2.83 ± 0.30 vs. 2.25 ± 0.41, P = 0.01, paired t-test) whereas the uptake in the uninvolved pancreas did not significantly change (SUV_mean_: 1.76 ± 0.43 vs. 1.94 ± 0.24, P = 0.41, in treated vs untreated timepoints, paired t-test) **(Figure 6D)**.

Histological evaluation of the available tumors and the available immediately adjacent non-cancerous pancreas tissues demonstrated higher collagen content (CPA) in the tumors compared with the adjacent pancreas (59 ± 9% vs. 17 ± 11%, P < 0.0001, in treated tumors vs. uninvolved adjacent pancreas respectively, unpaired t-test, two-tailed) **(Figure 6E)**. Response to treatment was confirmed on post-surgical histological tissue microscopic evaluations. A comparison of the initial core biopsy and resected tumor slides showed a significantly higher tumor CPA in the treated tissues (56 ± 6% vs. 32 ± 5%, P = 0.005, in 4 patients with available pre- and post-CRT tissues, paired t-test, and 59 ± 9% vs. 30 ± 9%, P = 0.0005, in all available tissues, n = 6 untreated and 5 treated, unpaired t-test) **(Figure 6F)**. There was a significant positive correlation between the tissue CPA and PET SUV_mean_ of PDAC and pancreas (R^2^ = 0.54, P = 0.0007) **(Figure 6G)**.

## Discussion

In this study, we demonstrated that ^68^Ga-CBP8 PET probe specifically targets and quantifies the dynamic changes of type I collagen in the PDAC ECM in both pre-clinical and clinical settings. The results of our first-in-human feasibility study suggest that ^68^Ga-CBP8 PET can provide a unique tool to non-invasively monitor treatment response in patients with PDAC. Fibrotic stroma is highly abundant in PDAC and is a major promotor of tumorigenesis, local invasion, and metastasis 18, 33. Type I collagen is the dominant component of tumor fibrosis and increases in response to chemotherapy 14. We previously demonstrated that higher collagen deposition in tumors following CRT is associated with longer overall and progression-free survival independent of other clinicopathologic and demographic variables in PDAC patients and therefore, changes in tumor collagen could be used as a predictor of clinical outcomes 14. Here, we validated ^68^Ga-CBP8 PET in multiple mouse models of human PDAC with different levels of sensitivity to chemotherapy and translated this technique to PDAC patients for the first time. ^68^Ga-CBP8 and analogous MRI probes where the Ga-68 is replaced with chelated gadolinium have been shown to detect, stage, and monitor fibrotic changes in animal models of cardiac, hepatic, and pulmonary fibrosis 23, 34-38. We recently showed the favorable biodistribution and dosimetry of ^68^Ga-CBP8 in healthy volunteers (NCT03535545) 26 and its ability to detect increased collagen in patients with pulmonary fibrosis 25, 27. The probe is currently under investigation in multiple clinical trials in different fibrotic conditions (NCT04485286, NCT03535545).

Given the overall poor prognosis of PDAC and high toxicity of standard chemotherapy regimens, more reliable imaging methods are required for differentiating the responders from non-responders and allowing clinicians with timely clinical management decisions to personalize the treatment strategies. Conventional CT and MRI used in daily clinical practice have limited abilities to provide such guidance as the changes in tumor morphology and size often lag behind the changes in their molecular signatures 10-13. Additionally, current standard methods for evaluation of PDAC response to neoadjuvant therapy such as radiographic response evaluation criteria in solid tumors (RECIST) and pathologic response grading, fail to predict survival in PDAC 10, 15, 39.

While changes in tumor metabolism on ^18^F-FDG PET have demonstrated valuable insights for determination of treatment response when combined with anatomical and/or biochemical assessments in pancreatic cancer, the utility of ^18^F-FDG PET has shown to be limited in multiple scenarios 40-42. Examples include variability of tumor ^18^F-FDG uptake in PDAC and other types of pancreatic tumors at baseline, challenges in detection of small lesions, difficulties in assessment of disease burden particularly in patients with peritoneal carcinomatosis, and confounding factors such as uptake in infectious and inflammatory conditions such as chronic pancreatitis, among others 40, 43, 44. Our study suggests that collagen PET could address these challenges for evaluation of response to treatment in PDAC patients.

We observed that collagen continuously increases within the ECM of chemotherapy-sensitive human PDAC xenografts as well as in the resected tissues from patients who responded to neoadjuvant CRT and that this change was accurately measured using collagen PET. Our histological findings align with previously published studies, where the collagen content showed a significant increase in pancreatic tumors treated with FOLFIRINOX or Gemcitabine 14, 20, 45. While some studies have suggested that a dense fibrotic tumor stroma at baseline can decrease the penetration and efficacy of medications 18, 19, others have shown that higher amounts of type I collagen and other fibrosis markers in untreated PDAC tissues correlate with longer patient survival 46-48. While we focused on quantifying the change in collagen deposition in this work, further investigation is necessary to determine whether the pre-treatment collagen and tumor desmoplasia are associated with neoadjuvant treatment response. The results of our study suggest that the change in tumor collagen due to therapeutics is a continuous and dynamic process, and a comparison of pre-and post-treatment collagen PET could be used as a non-invasive method for measuring this dynamic change and differentiating responders from non-responders. ^68^Ga-CBP8 PET has multiple advantages for PDAC over current investigational imaging techniques. While tumor enhancement patterns with iodinated contrast-enhanced CT and gadolinium-enhanced MRI have been suggested to differ in fibrotic tumors, a reliable evaluation method is not established 49. Limited number of studies have focused on changes in the b-values in restricted diffusion-weighted MRI and changes in tissue elasticity on elastography in fibrotic tumors, however, these methods still need to be validated for early assessment of treatment outcome 50, 51. Fibroblast activation protein targeting PET probes have shown superior accuracy for detection of PDAC in multiple clinical trials 52, 53, although the ability of this technique for evaluation of treatment response is under investigation. We anticipate that ^68^Ga-CBP8 PET can serve as a reliable and non-invasive quantitative surrogate of PDAC tumor fibrosis to guide management decisions for neoadjuvant therapy and assess treatment response. One of the major benefits of ^68^Ga-CBP8 PET is the possibility of simultaneous acquisition of information on the collagen content as a biomarker of response to treatment, in addition to the anatomic morphological changes assessed by CT or MRI. The rapid renal clearance and plasma stability of ^68^Ga-CBP8 and the 68 minute half-life of Ga-68 result in overall low radiation exposure to subjects allowing for serial PET imaging to monitor treatment response 54. Our study has multiple limitations. The preclinical studies were performed in subcutaneous human PDAC xenografts to avoid the interference of PET signal bleeding from the kidneys to the adjacent pancreas in small animals, and therefore, orthotopic mouse modes of PDAC could not be used. The mouse models of human PDAC with variable levels of sensitivity to chemotherapy were developed in immunocompromised mice and therefore, the interaction of host stroma and PDAC cells and lack of the immunocompetent tumor microenvironment may have affected the degree of fibrosis in the animal models. However, we observed a similar trend of collagen change in immunocompetent human experiments. While ^68^Ga-CBP8 PET was able to differentiate treatment responders from non-responders in animal models and resected human tissues, all the patients who completed pre- and post-treatment scans showed clinical and histological response to standard neoadjuvant treatment. Therefore, we were unable to evaluate the PET findings in patients resistant to standard chemotherapy. Further evaluation of ^68^Ga-CBP8 PET in larger scale clinical trials with a diverse patient population in both local and metastatic settings and with variable response to treatment is required to establish the utility and application of this technique in clinical practice.

In conclusion, our study shows that collagen PET provides a surrogate of treatment response in PDAC. This unique image-guided approach could significantly improve the quality of care by reducing the need for invasive procedures such as exploratory laparotomy and biopsy in the future. Collagen PET could also enable future clinical trials that use imaging of tumor fibrosis for early determination of treatment response and outcomes to accelerate drug development, particularly in the realm of anti-fibrotics. We anticipate collagen PET to improve cohort enrichment strategies and patient selection for trials in PDAC and other fibrotic neoplasms.

## Supplementary Material

Supplementary figures and table.

## Figures and Tables

**Table 1:** Study participants' inclusion and exclusion criteria

Inclusion Criteria	Age ≥ 18 y Ability to provide informed consent Histologically-proved diagnosis of PDAC Life expectancy of at least 3 months Standard-of-care baseline abdominal CT findings of borderline resectable or locally advanced PDAC within 3 months before pre-CRT PET visit Scheduled pre-CRT PET visit within 1 month prior to starting neoadjuvant CRT Scheduled surgical tumor resection within 1 month after post-CRT PET visit Available pre-surgical CT of abdomen within 1 month after completion of neoadjuvant CRT as part of routine clinical workup
Exclusion Criteria	Baseline CT report of metastatic PDAC History of reaction to MRI contrast agent(s) eGFR < 30 mL/min within 90 days of PET study visit Neoadjuvant treatment or prior radiation to upper abdomen before pre-CRT PET visit Acute pancreatitis within 6 weeks prior to pre-CRT PET visit Contraindications to MRI (claustrophobia, seizure disorder, metal, or implantable devices) Unable to lie comfortably on a bed inside the MR/PET scanner BMI > 33 (limit of the PET-MRI table) Recently received other radioisotopes with radiation exposure exceeding Radiology Department guidelines (i.e. 50 mSv in the prior 12 months) Pregnant or breastfeeding If determined by the investigator(s) to be clinically unsuitable for the study

PDAC: pancreatic ductal adenocarcinoma, CRT: chemoradiotherapy

**Table 2:** Study participants' information

#	Age (y)	Pre-CRT PET	Post-CRT PET	ECOG	Initial Disease	FOLFIRINOX Cycles (n)	Losartan	RT Fractions # (Gy)	Surgery	CA19-9 Pre-CRT	CA-19-9 Post-CRT
1	63	C	Declined	1	LA	8	No	28 + IORT 50.4	Whipple	210	19
2	64	C	C	0	BR	8	No	10 30	Whipple	1366	45
3	59	C	C	1	BR	8	No	28 50.4	Whipple	5018	48
4	65	C	C	0	BR	8	Yes	28 + IORT 50.4	Fibrosis only on intra-op Bx - GJ	122	26
5	49	C	Surgery Accelerated	0	LA	12	Yes	28 + IORT 50.4	Vascular involvement - GJ	2410	837
6	60	C	C	1	BR	8	No	28 50.4	Whipple	1921	10
7	64	C	C	0	BR	8	No	28 50.4	Whipple	377	17
8	58	C	Disease Progressed	1	BR	17	No	-	-	32632	9524

C: completed scan visit, LA: locally advanced, BR: borderline resectable, CRT: chemoradiotherapy, RT: Radiotherapy, IORT: intraoperative radiotherapy, ECOG: Eastern Cooperative Oncology Group performance status, GJ: gastrojejunostomy

## Abbreviations

PDAC: Pancreatic ductal adenocarcinoma
CRT: chemoradiotherapy
CT: Computed tomography
MRI: magnetic resonance imaging
PET: positron emission tomography
ROI: region of interest
%ID/g: percentage of injected dose per gram
ECOG: Eastern Cooperative Oncology Group
IHC: immunohistochemistry
CPA: collagen proportion area
HPLC: high-performance liquid chromatography
SD: standard deviation
Hyp: hydroxyproline
Pro: proline
PSR: Pcirosirius red
