# Supplementary material for: Collagen type I PET/MRI enables evaluation of treatment response in pancreatic cancer in pre-clinical and first-in-human translational studies
Source: Theranostics. 2024 Sep 9;14(15):5745–61. doi: 10.7150/thno.100116 (PMC11426233; doi:10.7150/thno.100116)
Supplement: Supplementary file 1 — Supplementary figures and table. [file thnov14p5745s1.pdf]

Supplementary Materials:

Table S1. ^{68}Ga-CBP8 administrated dosage and duration of PET

Subject #	Pre-CRT PET		Post-CRT PET	
	Dosage (MBq)	PET Duration (min)	Dosage (MBq)	PET Duration (min)
**1**	122	51	-	-
**2**	185	90	220	90
**3**	281	90	131	90
**4**	232	90	133	60
**5**	167	90	-	-
**6**	195	47	332	30
**7**	173	60	128	30
**8**	205	60	-	-

CRT: chemoradiotherapy

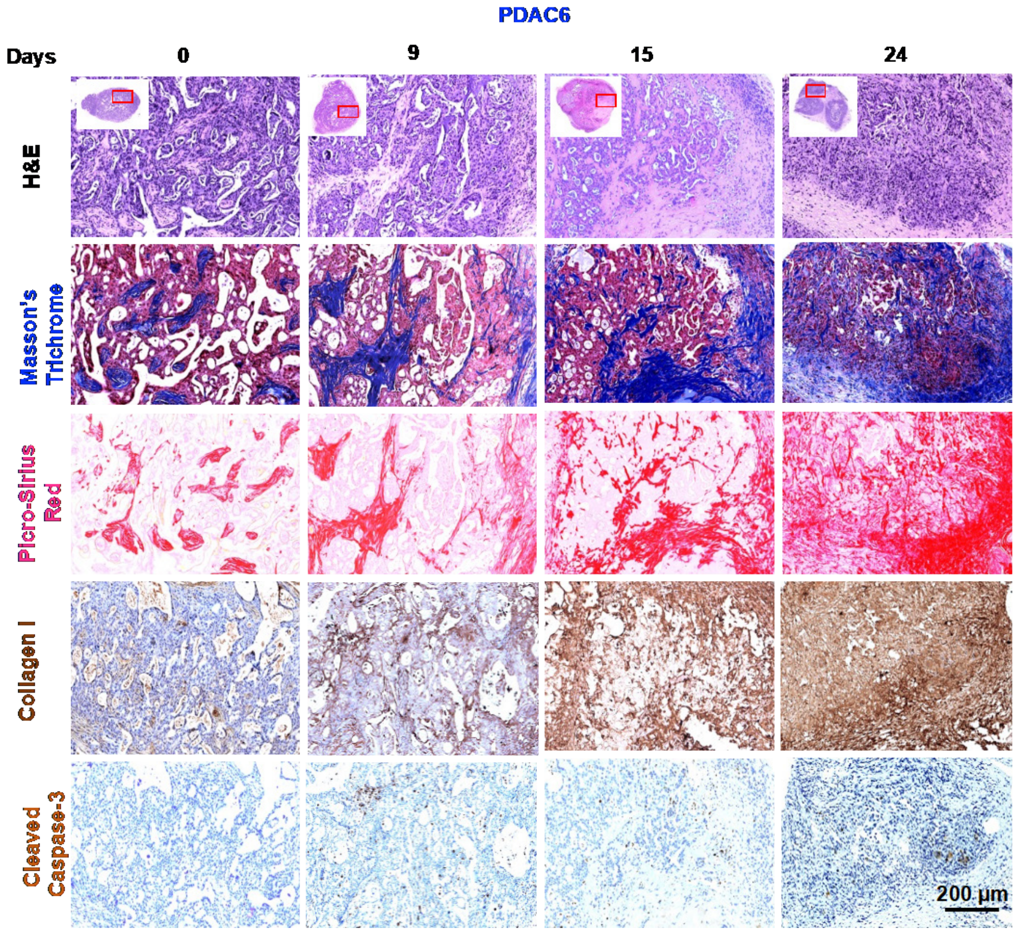

Figure S1. Representative histological images of the PDAC6 tumors show a continuous decrease in tumor cellularity, and increased deposition of collagen and cleaved caspase-3 as a marker of apoptosis. PDAC6 cells were obtained from Dr. David Ting's laboratory. Scale bar: 200 µm

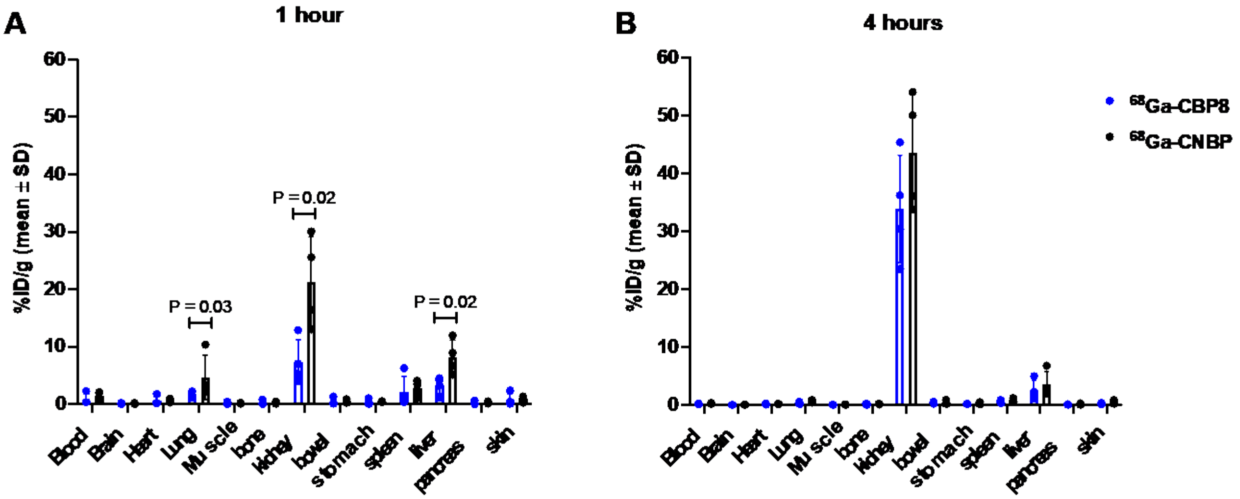

Figure S2. Ex vivo biodistribution analysis of type 1 collagen binding ^{68}Ga-CBP8 and control linear non-binding ^{68}Ga-CNP probe in non-tumor bearing male nude mice at 1 h and 4 h post-injection. There were no significant differences in organ concentration between ^{68}Ga-CBP8 and ^{68}Ga-CNP at any time, except in the lung, kidney, and liver at 1 h post-injection.

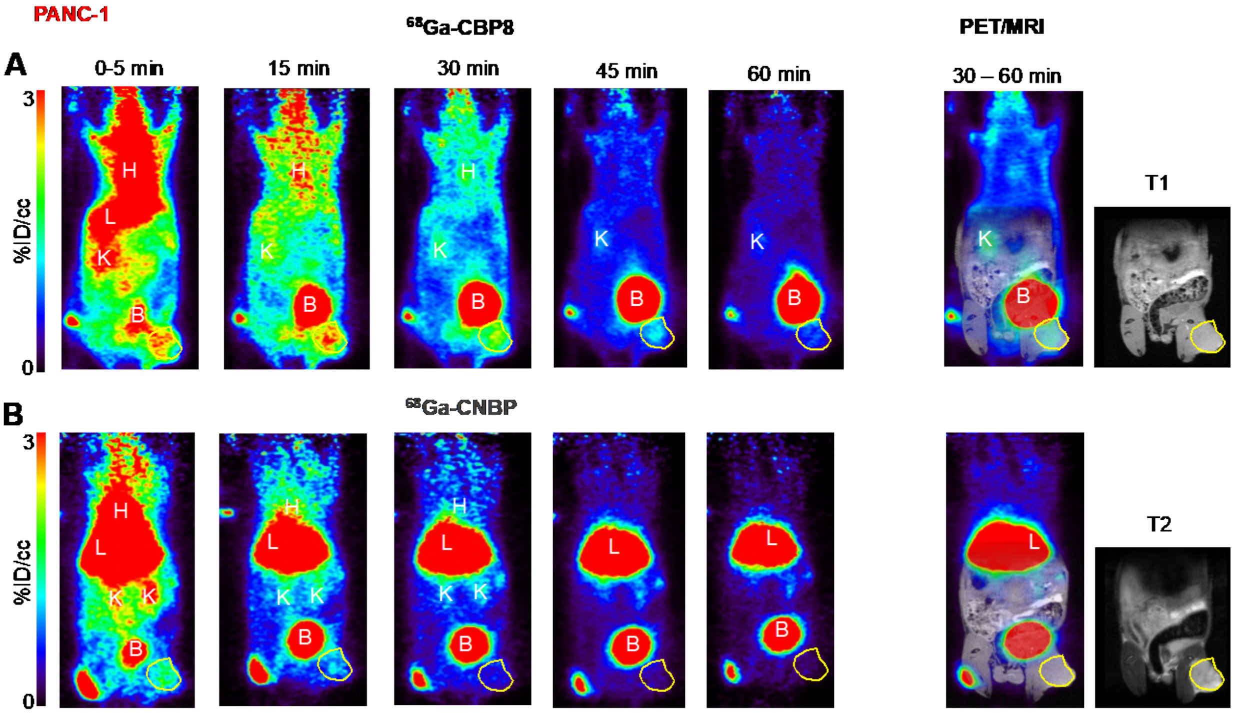

Figure S3. Representative coronal PET images of PANC-1 tumor-bearing mice 0-60 min after intravenous administration of the collagen-specific ^{68}Ga-CBP8 **(A)** and control ^{68}Ga-CNBP **(B)** probes (tumors shown in yellow circle). The 30-60 min post-injection was selected as the optimal time for static reconstruction and shorter scans in the subsequent experiments of this study. Fused coronal PET and T1 weighted MRI are shown for each probe. T1 and T2 weighted MR images of the same mouse were used for localization purposes. H: Heart, L: Liver, B: Bladder, K: Kidney

PDAC6

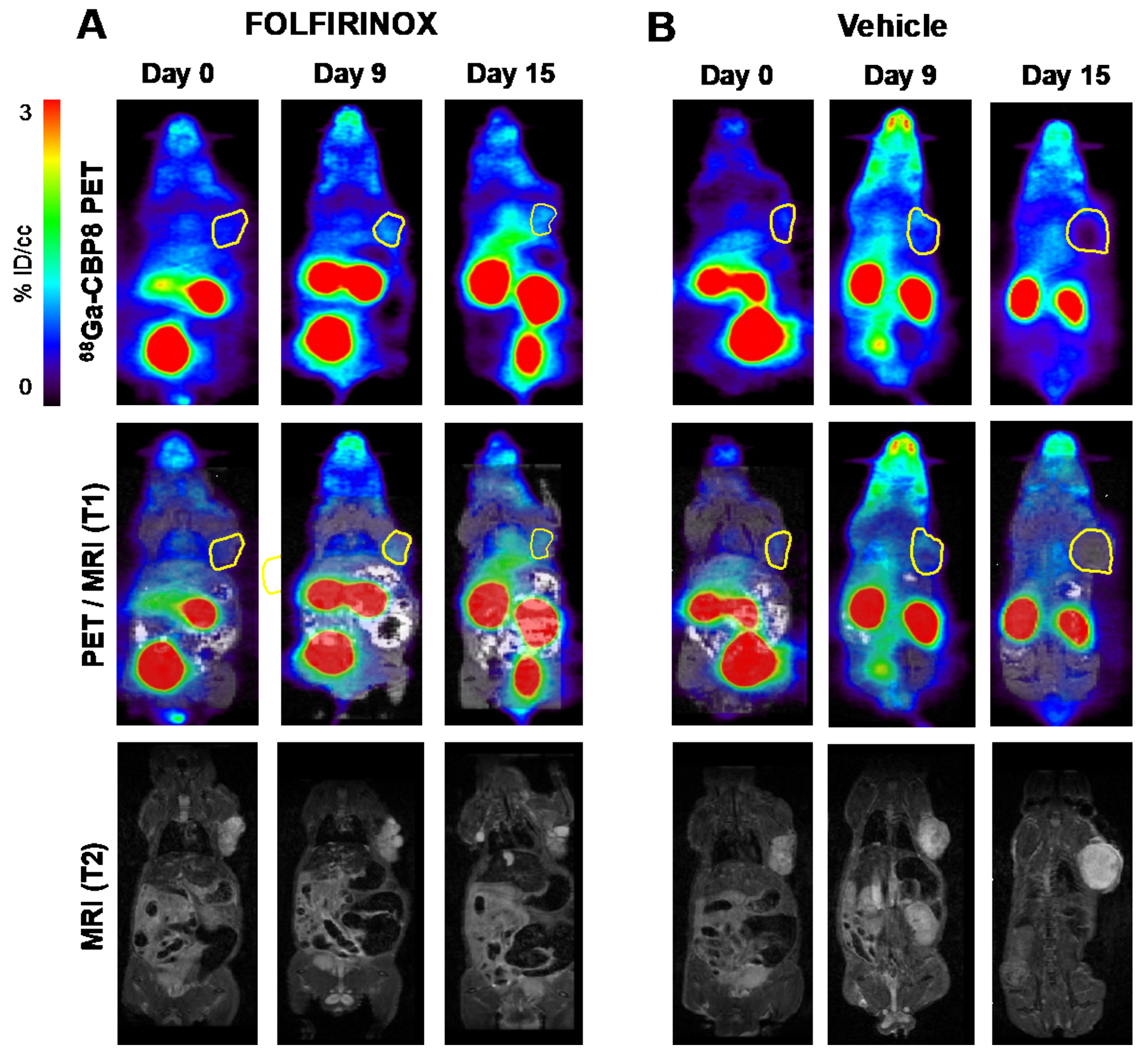

Figure S4. Representative Coronal ^{68}Ga-CBP8 PET, fused PET with fat saturated T1-weighted MRI and T2-weighted images of mice implanted with PDAC6 (yellow circle) treated with FOLFIRINOX **(A)** or vehicle **(B)**. PET images were acquired at 30-60 min post-injection.
